# A Structural Approach to Anti-Virulence: A Discovery Pipeline

**DOI:** 10.3390/microorganisms9122514

**Published:** 2021-12-04

**Authors:** Michael McCarthy, Monica Goncalves, Hannah Powell, Blake Morey, Madison Turner, Allan Rod Merrill

**Affiliations:** Department of Molecular and Cellular Biology, University of Guelph, Guelph, ON N1G 2W1, Canada; mmccar07@gmail.com (M.M.); Mgonca06@uoguelph.ca (M.G.); hpowell@uoguelph.ca (H.P.); Blakemorey56@gmail.com (B.M.); mturne09@uoguelph.ca (M.T.)

**Keywords:** mono-ADP-ribosyltransferase toxins, anti-virulence agents, bacterial toxins, protein crystallography, flavo-noids, natural products, honey bee diseases, American Foulbrood, drug discovery

## Abstract

The anti-virulence strategy is designed to prevent bacterial virulence factors produced by pathogenic bacteria from initiating and sustaining an infection. One family of bacterial virulence factors is the mono-ADP-ribosyltransferase toxins, which are produced by pathogens as tools to compromise the target host cell. These toxins are bacterial enzymes that exploit host cellular NAD+ as the donor substrate to modify an essential macromolecule acceptor target in the host cell. This biochemical reaction modifies the target macromolecule (often protein or DNA) and functions in a binary fashion to turn the target activity on or off by blocking or impairing a critical process or pathway in the host. A structural biology approach to the anti-virulence method to neutralize the cytotoxic effect of these factors requires the search and design of small molecules that bind tightly to the enzyme active site and prevent catalytic function essentially disarming the pathogen. This method requires a high-resolution structure to serve as the model for small molecule inhibitor development, which illuminates the path to drug development. This alternative strategy to antibiotic therapy represents a paradigm shift that may circumvent multi-drug resistance in the offending microbe through anti-virulence therapy. In this report, the rationale for the anti-virulence structural approach will be discussed along with recent efforts to apply this method to treat honey bee diseases using natural products.

## 1. Introduction

Since their serendipitous discovery by Alexander Fleming, antibiotics have been the basis in modern medicine for the treatment of bacterial diseases because of the life-saving compounds produced by Florey during World War II. However, the emergence of multidrug resistance bacteria has threatened to unravel this approach to human and animal health. The global spread of resistant microbes in all areas of disease treatment has left the antibiotic drug pipeline unable to meet demand since compounds are metabolized by voracious multi-drug resistant organisms [1,2,3]. The situation begs for new strategies to combat microbial pathogens [2,4,5,6,7].

A compelling, innovative, and alternative approach to antibiotic therapy is the anti-virulence or anti-infective strategy that involves targeting virulence-associated rather than survival/fitness-relevant traits in the offending pathogen [8,9,10,11,12,13]. Thus, the “anti-infective” agent/drug interferes with the pathogen’s ability to cause disease [14,15]. One avenue for this approach is to target bacterial toxins using this powerful method [9,16,17,18,19]. This approach involves the use of patho- or virulence blockers developed specifically to bind bacterial virulence factors with high affinity, neutralizing or reducing the virulence of the offending pathogen [17,20,21]. Anti-virulence compounds offer significant advantages over conventional antibiotics. Firstly, these agents are directed towards specific mechanisms in the offending pathogen that promote infection or harm host cells rather than targeting an essential growth/metabolic factor. Disarming microorganisms of their virulence properties without threatening their survival offers lower selection pressure, reducing the risk of drug-resistant mutations. Secondly, virulence-specific therapeutics avoids the collateral damage on the host microbiota associated with current antibiotics. Other possible advantages to the anti-virulence approach include limited off-target effects due to the unique mechanism of the mART toxin action and targeting a conserved motif within a family of proteins provides a potential to target multiple pathogens with a single small molecule compound. Thus, the anti-virulence approach has the potential for a paradigm shift in the development and treatment of bacterial diseases and infections. This novel approach makes pathogenic bacteria less virulent by neutralizing their weaponry, thereby giving the host’s immune system time to clear the pathogen. Additionally, the weakened pathogen is more susceptible to antibiotic therapy [15].

Bacterial toxins of all types are a common infection tool produced by bacterial pathogens to cause disease. These factors facilitate the infection process by the pathogen and are often key elements in disease development. Compounds, known as anti-virulence agents, are designed to interfere or block the toxin machinery and associated molecular mechanisms and hold promise to thwart bacterial pathogens. 

**Figure 1:**
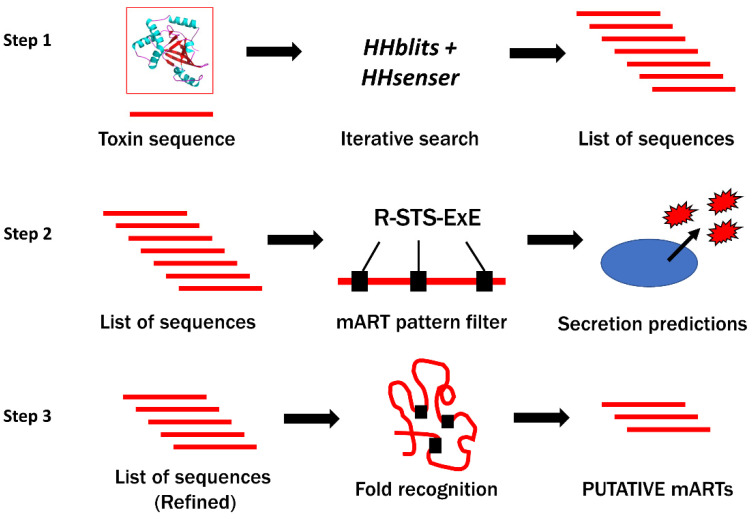
Summary of mART toxin discovery pipeline. Step 1: Known mART toxin sequences are used (input) to provide several thousands (10,000–20,000) of candidate toxin sequences arising from iterative search tools such as HHblits and HHsenser. This mining strategy is analogous to a large-scale BLAST search that cater to protein homologs with low sequence identity [32,33]. Step 2: Sequences are then subjected to pattern-based filters, including searches for the conserved mART motif, followed by predictions for transmembrane domains (unwanted), and for secretion signal peptides or other indicators of non-classical secretion. Step 3: Finally, consensus fold recognition analyses determine whether the remaining sequences may yield a mART-like fold by generating homology models. The final list contains putative mART toxin sequences for experimental testing in a yeast growth-deficiency assay to validate computer predictions [34] [taken from Tremblay et al. *Toxins* 12: 792, 2020].

A growing collection of bacterial toxins that serve as high profile targets for anti-virulence intervention is the mono-ADP-ribosyltransferase (mART) family. Family members covalently modify specific host macromolecules as an “on—off” switch often producing a unique pathology. Notably, diphtheria toxin (DT) is a classic example of a mART toxin that is the sole cause of a disease (diphtheria) by modifying ribosomal elongation factor 2 (eEF2), terminating protein synthesis, and leading to apoptosis [22]. The cellular targets for mART toxins are often key regulators of cell function and include (i) GTPases, (ii) actin, (iii) kinase regulators, (iv) elongation factors, and (v) RNA-recognition motifs, and even DNA (target genes) [22,23,24,25,26,27,28]. We developed a data-mining strategy based on fold-recognition methods [29,30,31] that unearths new mARTs as targets for disease intervention using the anti-virulence or anti-infective approach (Figure 1).

Our efforts to date have led to the discovery of 30 new mARTs with rigorous characterization of several toxins accompanied by more than 50 crystal structures, including ExoA (*P. aeruginosa*) [35,36,37,38,39,40,41,42,43,44,45,46,47], Cholix (*V. cholerae*) [34,48,49,50,51,52,53,54,55], Certhrax (*B. cereus*) [56], Photox (*P. luminescens*) [57,58], VahC/VsdC (*A. hydrophila*) [59], C3larvinA, Plx2A (*P. larvae*) [60,61,62,63], Vis (*V. splendidus*) [64], Scabin (*Streptomyces scabies*) [23,65,66,67], and Vorin (*E. amylovora*) [31].

## 2. Materials and Methods

### 2.1. Recombinant Plx2A

The honey bee toxin, Plx2A was expressed in BL21 λDE3 *E. coli* cells as a recombinant His_6_-tagged protein (pET-28+ vector) and was purified by immobilized metal-affinity chromatography (IMAC) followed by size-exclusion chromatography as previously described [61]. For enzymatic assays, the His_6_-tag was not removed, and it was shown that the tag did not affect enzyme activity. 

### 2.2. Small Molecule Inhibitors of Plx2A

The synthetic inhibitor, M3, was purchased from Molport (Riga, Latvia) and the flavonoid inhibitors were commercially obtained from ChemFaces Biochemical Co., (Wuhan, China). All inhibitors were stored at 4 °C at 10 mM dissolved in 100% DMSO. 

### 2.3. Glycohydrolase (GH) Activity

Plx2A GH activity was measured in a Cary Eclipse fluorescence spectrometer (Agilent Technologies Inc., Mississauga, ON, Canada) using the fluorescent NAD^+^ analogue, etheno-NAD^+^ (ε-NAD^+^) as the substrate for the hydrolysis activity of the enzyme. Measurements were conducted at 25 °C in the presence of 5 μmol/L Plx2A in GH reaction buffer (20 mmol/L Tris, 50 mmol/L NaCl, pH 7.9) with a final reaction volume of 80 μL in disposable UVette cuvettes (Eppendorf, ON, Canada). The excitation wavelength was 305 nm and emission were set at 405 nm with 5 nm bandpasses for both excitation and emission. Triplicate reactions were monitored for 10 min and the initial slope of the reaction was recorded. An ε-AMP standard curve was generated to convert fluorescence units per min to the concentration rate of ε-ADP-ribose product formed by the enzyme. A Michaelis–Menten curve was produced by fitting the data to the hyperbolic equation in OriginPro ver8 software (OriginLab Corporation, Northhampton, MA, USA).

### 2.4. Inhibitor Binding to Plx2A

The binding constants, K_D_, for inhibitors with Plx2A were determined by measuring the quenching of the intrinsic tryptophan fluorescence of the protein using a Cary Eclipse fluorescence spectrophotometer. Samples were excited at 295 nm and emission was monitored at 340 nm with 5 nm bandpasses. Plx2A at 1.25 μmol/L in 50 mmol/L Tris-HCl, pH 8, and 100 μmol/L NaCl was titrated with inhibitors (0–500 μmol/L) and the fluorescence intensity was monitored with 15 s integration time. Assays were performed in triplicate and the data were corrected to account for the increase in volume upon addition of inhibitor. A blank titration with *N*-acetyltryptophanamide (NATA) was performed to correct for inner filter effects. Data were analyzed using OriginPro ver8 software to determine the binding constants.

### 2.5. IC_50_ and K_i_ Determination

Kinetic inhibition assays were conducted to determine the IC_50_ values for flavonoid inhibitors and the experiments were conducted on a Cary Eclipse spectrophotometer with an excitation of 305 nm, emission wavelength of 405 nm and bandpasses of 5 nm. Plx2A (5 μmol/L) was mixed with 250 μmol/L ε-NAD^+^, at various inhibitor concentrations in GH buffer. Triplicate reactions at 25 °C were monitored for 10 min intervals and then the initial slope was calculated for each trace. The kinetic data were fit to a Michaelis–Menten function generated in OriginLab Pro ver8 software to determine the IC50 value. The mechanism of flavonoid inhibition was determined for Quercetin as the model compound and entailed collection of full Michaelis–Menten datasets in the presence of 0, 10, 20, 30, and 40 μmol/L of inhibitor in a final DMSO concentration of 15% (*v*/*v*). Plx2A GH activity is not affected by DMSO in the reaction buffer until 20% (*v*/*v*). The inhibition data were transformed to the Lineweaver–Burk function and plotted to determine the inhibition pattern. Secondary plots were then generated to calculate the K_i_ value, which represents the Quercetin binding constant for Plx2A.

## 3. Results

### 3.1. Anti-Virulence Approach

#### 3.1.1. Virtual Screens

**Figure 2:**
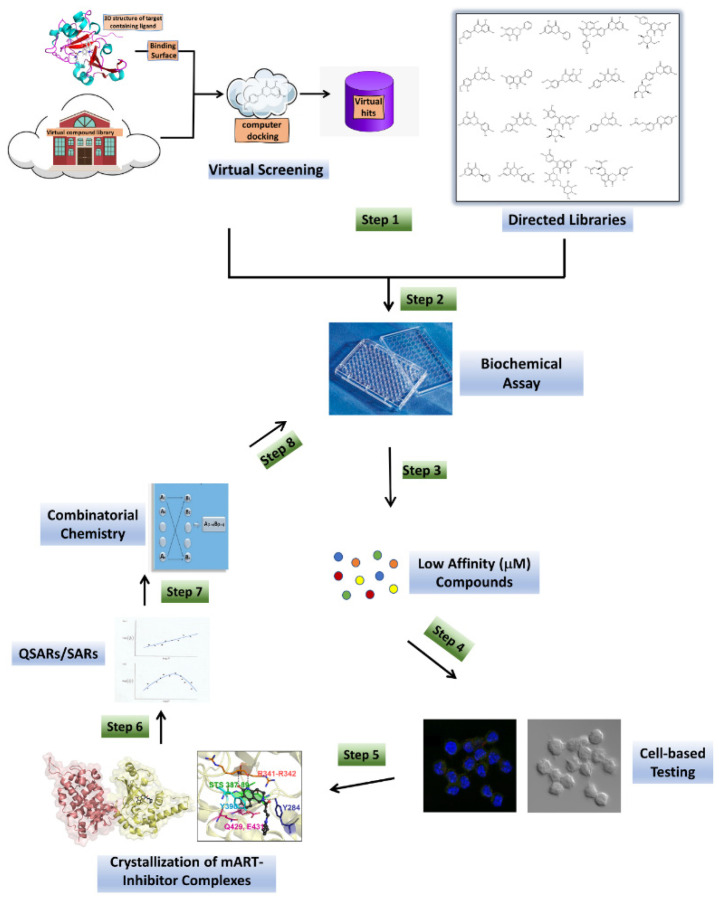
Anti-virulence development pipeline for mART toxins. mART inhibitors arise from two major sources, namely, virtual screening using a high-resolution mART structure of a toxin-inhibitor complex and “hand-picked” directed libraries (Step 1). Candidate compounds are then subjected to a Biochemical Assay (Step 2), producing a library of active, low-affinity compounds (μmol/L affinity for binding site), which are then chosen for cell-based testing (Step 3). Promising inhibitor compounds are then tested for efficacy to protect the corresponding target host cells from the mART toxin (Step 4). The most efficacious compounds (leads) are co-crystallized (Step 5) with their cognate toxin followed by lead optimization through Structure–Activity Relationships/Quantitative Structure–Activity Relationships methodology (Step 6). Combinatorial Chemistry methods are then used to prepare derivatives of the best compounds (Step 7), which are then re-tested in the Biochemical Assay (Step 8) and the cycle of development continues until higher-affinity compounds (nmol/L or greater) are developed.

One facet of our approach is to use relevant high-resolution crystal structures as the template models for virtual screening (Figure 2). The PDB structure is prepared for molecular docking using appropriate software (OpenEye, http://www.openeye.net/products/software/ (accessed on 5 May 2017); Schrodinger software suite, https://www.schrodinger.com/ (accessed on 6 June 2018) run on a super-computer network [68,69]. Virtual screens yield a list of potential “hits” against the anti-virulence drug target (receptor) and can produce valuable contributions in “hit-and lead-compound” discovery [70,71,72] with promising results [73,74,75,76,77,78] using iota toxin in complex with NADH (1GIQ:C2-like) [59,64] and C3bot1 toxin (2C8A:C3-like) [60]. Online compound libraries such as the ZINC Drugs NOW set of 6.5 million drug-like compounds https://zinc12.docking.org/subsets/drugs-now (accessed on 6 June 2018) serve as a starting source of compounds for docking experiments with methods as described [60]. Ultimately, only a small, manageable subset of compounds (usually less than 100) was selected for testing as inhibitors of mART toxin enzyme and cytotoxic activity [59,60,64].

#### 3.1.2. Directed Libraries

We recently chose flavonoid compounds as our directed library source of small molecules [79] to complement the virtual screen approach. Flavonoids are natural products produced as plant secondary metabolites and are polyphenols that feature two phenyl rings bridged with a pyran or pyranone ring (see Table 1). Importantly, flavonoids show diverse biological activities, including antibacterial activity [80], inhibitors of proteins in cells, or their membranes and membrane perturbation activity [81,82]. Initially, a flavonoid library of 1200 compounds were sourced as potential anti-virulence agents against mARTs, and finally, 20 compounds were chosen for experimental testing based on several filter criteria, including literature reports of antimicrobial activity. 

#### 3.1.3. Biochemical Assay

Initial testing of the inhibitory activity against mART toxins involves an enzymatic assay, since these virulence factors are bacterial enzymes that cleave the NAD^+^ substrate and transfer ADP-ribose to the macromolecule substrate. However, most mARTs also show a secondary activity known as glycohydrolase (GH) that involves direct hydrolysis of NAD^+^ in the absence of a second substrate. A fluorescence-based plate-reader enzyme assay is often used to determine the inhibitor properties (IC_50_, K_i_ values) of the selected compounds (Figure 2). A fluorescence-based binding assay for lead compounds with the target mART is also established based on quenching of intrinsic fluorescence upon enzyme-inhibitor association to provide a measure of binding affinity (K_D_) [9].

#### 3.1.4. Cell-Based Testing

Compounds with low micromolar (μmol/L) efficacy from the biochemical assay step (Figure 2) are then tested in a cell-based system that is specific for the mART-producing pathogen (Figure 2). Effective compounds are identified as those agents that provide protection of mART-intoxicated cells at low micromolar doses while not causing cellular cytotoxicity. This assay provides a direct measure of the dose–response (ED_50_ value) and cellular toxicity (TD_50_ value) of the inhibitor compounds. The best compounds are those with the highest therapeutic index (TD_50_/ED_50_) as a measure of inhibitor potential. 

#### 3.1.5. Crystallization of mART-Inhibitor Complexes

The co-crystal structures of mARTs with the best lead compounds are pursued to provide important structural biology insights into the location, nature, and chemistry of the inhibitor-binding sites (Figure 2). The details of the toxin-inhibitor complexes with the various mARTs provides new insights into the structural features/differences of the binding pockets for mART toxins. These structural data also provide the basis for further rationale inhibitor design to iteratively evolve/design lead hits into bona fide drug candidates.

#### 3.1.6. SARs/QSARs and Combinatorial Chemistry

Rational inhibitor design and improvement involves SARs and QSARs studies to iteratively evolve lead hits into high-affinity drug candidates (Figure 2). Lead compounds identified in virtual screens or from “directed libraries” that show promise in enzyme and mammalian cell assays are further developed with respect to binding potency and pharmacokinetic properties. These compounds are investigated initially through a combination of modeling and docking studies using the target crystal structure to see which groups are essential for target binding, while at the same time introducing water-solubilizing groups with an eye to keeping the pK_a_ values for compounds between 5–6. New second- and third-generation analogues can be synthesized using the unique microwave-assisted, continuous flow synthesis platform [83,84,85] (Figure 2). Conventional Lipinski parameters are used to guide analogue design [86], which are obtained using Drug Discovery software. As new candidates arise from the screen efforts, they are brought through the same optimization process and the cycle continues until high-affinity compounds are achieved.

### 3.2. Anti-Virulence Agents against American Foulbrood

#### 3.2.1. Paenibacillus Larvae 

Honey bee (*Apis mellifera*) beekeepers are faced with multiple challenges globally when maintaining bee colonies for pollination and honey production [87]. American Foulbrood is a bacterial disease that afflicts the brood and is responsible for huge economic losses in the apiary [88,89]. The disease is caused by *Paenibacillus larvae*, a Gram-positive, rod-shaped bacterium that forms spores that can remain viable for decades [90]. The ERIC genotypes of *P. larvae* strains are named after their enterobacterial repetitive intergenic consensus sequences and exist as five strains, where only two are currently environmentally active [91]. ERICI is found worldwide and is the slowest-killing phenotype, which helps to avoid the housekeeping of nurse bees, while ERICII is the most virulent species that occurs only in Europe and can infect an entire colony within a week [92]. Several virulence factors produced by *P. larvae* have been identified and their biology and role in pathogenicity have been reported [88,93]. These factors include two mART toxins, Plx1 and Plx2, shown to be important ERICI virulence factors [94]; Plx2A was recently characterized and crystallized [61]. However, to date, only a few of the *P. larvae* virulence factors have been targeted for an anti-virulence strategy [9,61,95].

#### 3.2.2. C3larvin Toxin

C3larvin was identified by a bioinformatics approach as a mART in the C3 toxin subgroup and putative virulence factor from *P. larvae*. Cytoplasmic expression of C3larvin in a yeast model system revealed that it is highly cytotoxic [60]. C3larvin catalytic variants (Q155A, E157A, Q155A/E157A) with reduced or no mART enzymatic activity, were not toxic to yeast (eukaryotic model), revealing that C3larvin was a mART toxin with enzymatically driven cytotoxicity in a eukaryotic host. C3larvin showed mART enzyme activity and labeled Asn41 on RhoA, as seen in the C3 subgroup [60]. Surprisingly, C3larvin did not enter target macrophages unlike other C3 subgroup members, and it was determined that C3larvin possessed a truncated helix 1. It is now appreciated that it is only functional in a unique *P. larvae* strain of the MLST sequence type 9 in ERICIII/IV [95]. Two crystal structures of C3larvin revealed that it is indeed a C3 subgroup member with a shortened helix 1 (PDB:TR5; PDB:5DZQ), although the protein is not functional in bee larval assays [62]. 

An inhibitor, M3, of C3larvin mART enzymatic activity was identified by a virtual screen [64]. The methane sulfonamide derivative inhibited the enzyme with a K_i_ = 11 μmol/L and was the first inhibitor reported for the mART C3 subgroup [60]. M3 is unusual as an inhibitor of mART toxins because it possesses an adenine ring linked to substituted piperidine. M3 was also shown to be an effective inhibitor against Vis toxin from *Vibrio splendidus* and C3bot1 from *Clostridium* botulinum [64]. A molecular mechanics/molecular dynamics approach suggested that M3 competes with the adenine ring of the NAD^+^ substrate, but verification awaits a high-resolution co-crystal structure [60].

#### 3.2.3. Plx2A Toxin

**Figure 3:**
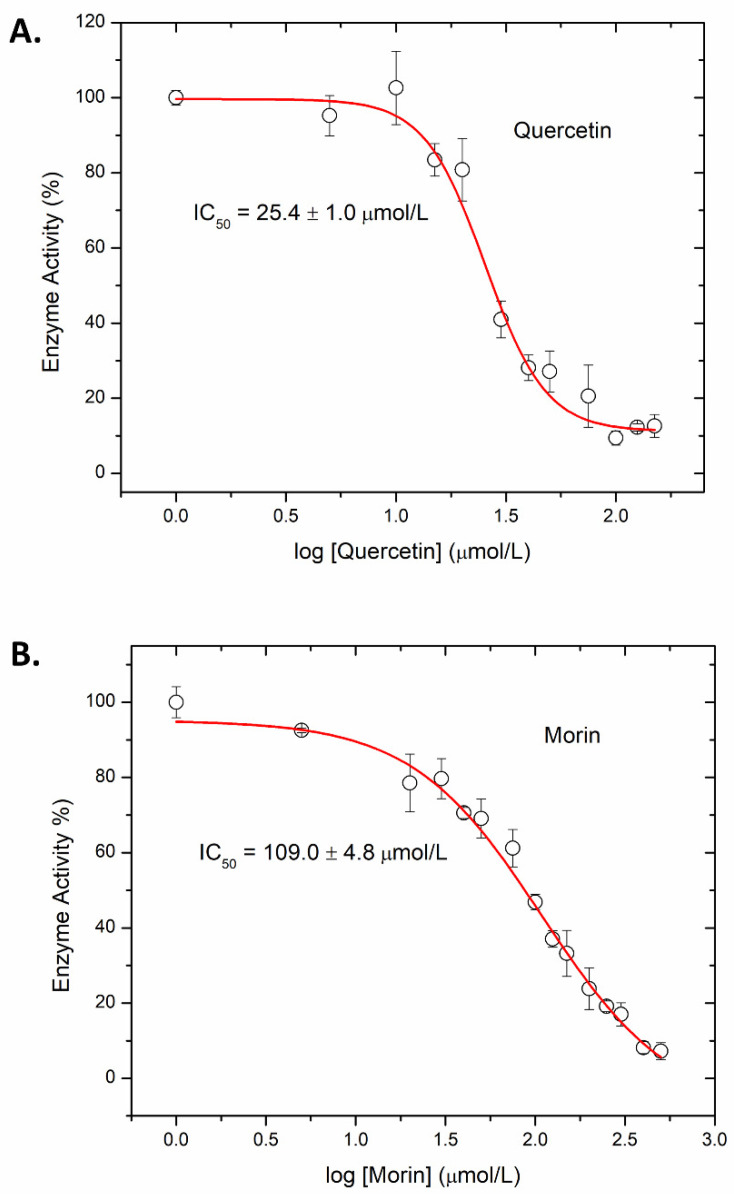
IC_50_ dose–response curves (representative) for Plx2A GH activity. The concentration of inhibitor that reduced the Plx2A GH enzyme activity by 50% (IC_50_ value) was determined as described in the Material and Methods section. (**A**) Quercetin dose–response curve, (**B**) Morin dose–response curve. Each data point was collected in triplicate and averaged (mean ± S.D.) and the experiment was repeated three times. Eight compounds showed strong inhibitory activity against Plx2A GH activity (Table 1) with four flavonoids exhibiting IC_50_ values less than 50 μmol/L.

*P. larvae* ERICI virulence largely depends upon two AB toxins, Plx1 and Plx2. Plx2A is the enzymatically active A-subunit of Plx2 and is a virulence factor in honey bee larva [61,94,95]. Plx2A targets host cell RhoA and inhibits cytokinesis in an insect model system [61]. Recently, it was shown that the synthetic compound M3 inhibits the enzymatic function of Plx2A [9]. Additionally, several flavonoids were found to be effective inhibitors of Plx2A catalytic function including baicalein and acacetin. A flavonoid library of 1200 compounds were filtered based on several criteria, including structural similarities to known mART inhibitors, literature reports of microbial activity, and chemical stability to produce a directed library of 20 compounds for experimental testing against Plx2A activity [9]. Figure 3 shows the typical dose–response plots for the activity of Plx2A against both Quercetin and Morin inhibitor concentrations. The midpoints of these curves represent the IC_50_ values, which are the enzyme activity at 50% of the uninhibited levels. Eight compounds showed strong inhibitory activity against Plx2A GH activity, with four flavonoids exhibiting IC_50_ values less than 50 μmol/L (Table 1).

**Figure 4:**
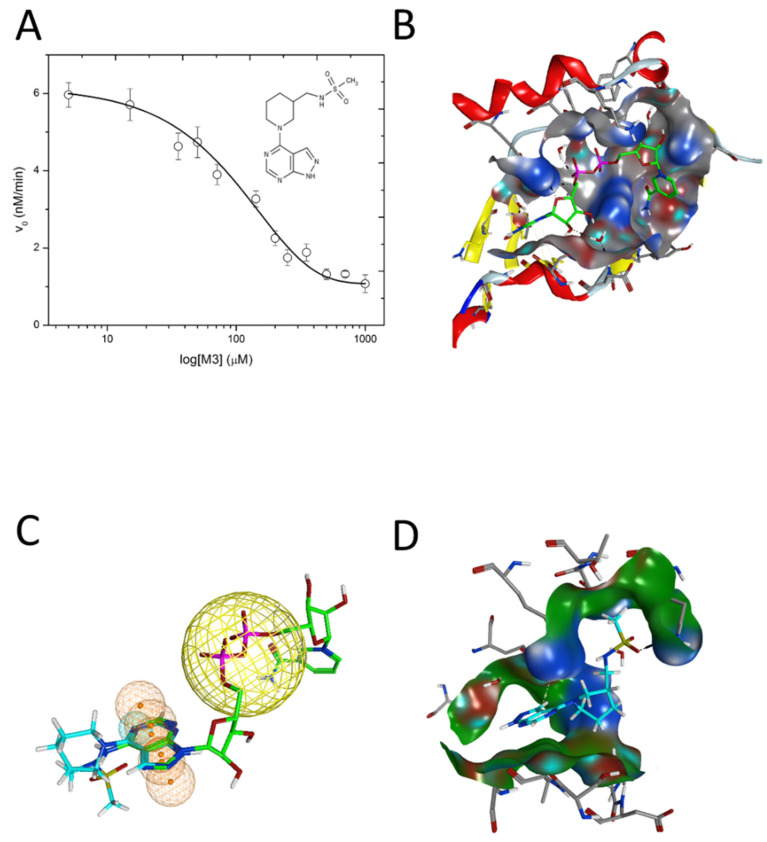
Inhibition of C3larvin GH activity. (**A**) Dose–response curve for the M3 inhibitor on C3larvin activity. Reduction in GH activity upon increasing doses of M3 inhibitor as described in the Materials and Methods section and the IC_50_ value was calculated from the data. Error bars, S.D from at least three experiments. Inset to (**A**): M3 inhibitor structure, *N*-[(1-[1H-pyrazolo[3,4-d]pyrimidin-4-yl]piperidin-3-yl)methyl]methanesulfonamide. (**B**) C3larvin/Plx2A (gray surface) based on the NAD^+^ active conformation (green C-atoms). (**C**) Pharmacophore model for C3larvin. Modeled active NAD^+^ (green C-atoms) on C3larvin, with M3 (cyan C-atoms) manually superposed with the adenine ring-system, to reveal common features. The orange spheres/mesh show the NAD^+^ adenine pharmacophore definition and the large yellow sphere is an anion-center feature. (**D**) Docked poses of M3 (cyan C-atoms), based on the pharmacophore definition with an induced fit (flexible) receptor [taken from Krska et al. *J. Biol. Chem*. 290, 1639–1653, 2015].

In addition, the binding affinity of the inhibitors for Plx2A was measured directly from the quenching data for the intrinsic tryptophan fluorescence of the protein upon titration with inhibitor (Table 1). The binding affinity (K_D_ values) ranged from 13.6 ± 1.5 μmol/L for Baicalein to 40.6 ± 4.7 μmol/L for Chrysin, indicating that the best flavonoid inhibitors bind with good affinity to the Plx2A protein. Previously, we proposed that the synthetic inhibitor, M3, which consists of an adenine ring linked to piperidine ring with a sulfated amine side-chain (see the structure in Table 1), competes with the adenine portion of NAD^+^, unlike most known mART inhibitors [60]. Most mART inhibitors compete with the nicotinamide portion of the NAD^+^ substrate [23,36,48,55,56,64]. Our results are based on an MM/MD approach of the C3larvin X-ray structures (PDB:4TR5; 5DZQ) and a ligand pharmacophore in the C3larvin active-site (nearly identical C3toxin with Plx2A). The two nucleotide bases comprising the NAD^+^ substrate each dock within separate subsites in the Plx2A active-site pocket and the nicotinamide pocket is much deeper and more nonpolar than the adenine-binding pocket. According to our analysis, M3, as a substrate inhibitor, likely competes with the adenine moiety of NAD^+^, with Lys52, Asn55, and Arg59 forming strong interactions with M3 (Figure 4) [60].

**Figure 5:**
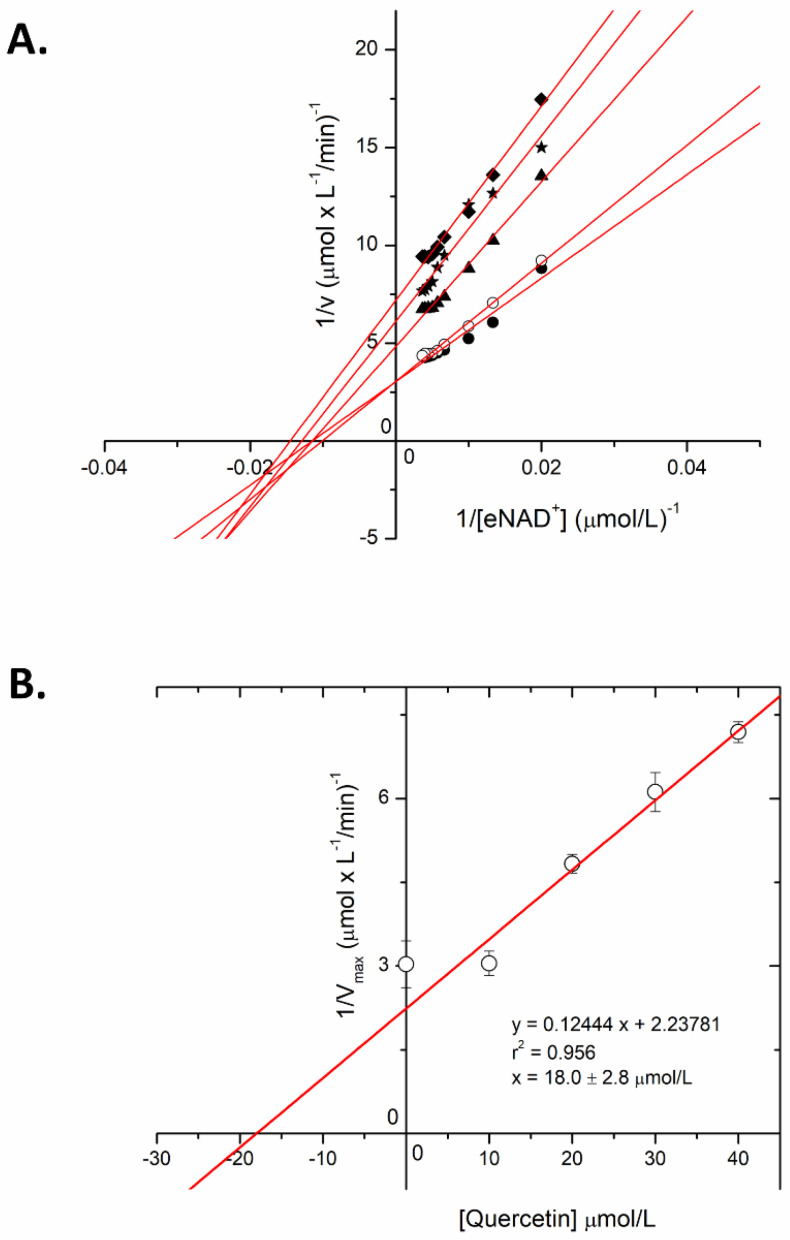
Inhibition of Plx2A GH activity with Quercetin. (**A**) Lineweaver–Burk plots of Quercetin GH activity inhibited by Quercetin. The assays were described in the Materials and Methods in the presence of 5 μmol/L Plx2A and a gradient of ε-NAD^+^ concentrations (0 μmol/L–400 μmol/L) in GH buffer (50 mmol/L NaCl, 20 mmol/L Tris), pH 7.5, at 25 °C with 15% DMSO in the solution to provide solubility to the flavonoid. (**B**) Secondary plot based on the non-competitive inhibition pattern for Quercetin against Plx2A GH activity. The intercept on the abscissa revealed the K_i_ value (18.0 ± 2.8 μmol/L). The experiments were conducted in triplicate with means calculated ± SD.

Michaelis–Menten kinetic analysis of the Plx2A GH enzyme activity was conducted to reveal the mechanism of flavonoid inhibition for Plx2A with Quercetin as the representative member of the flavonoid inhibitor series (IC_50_, 24.3 ± 0.8 μmol/L; Table 1). Michaelis–Menten data were collected for the GH activity of Plx2A at four concentrations of Quercetin inhibitor and the data were plotted as Lineweaver–Burk transformations to reveal the inhibition signature for the flavonoid series. The data lines for the control (no inhibitor) and the various inhibitor concentrations showed the signature pattern for non-competitive inhibition with the lines intersecting on the abscissa (Figure 5A). The V_max_ for the GH activity of Plx2A diminished in accord with the Quercetin inhibitor dose and the K_M_ value remained relatively constant over the course of the inhibitor doses (K_M_ = 84.0 ± 11.3 μmol/L). A secondary plot of the Quercetin inhibition kinetic data allowed for the calculation of the K_i_ value (Quercetin binding constant) of 18.0 ± 2.8 μmol/L (Figure 5B). This value agrees well with the 24.3 μmol/L for the IC_50_ value determined for the Quercetin with the Plx2A enzyme (Table 1).

## 4. Discussion

### 4.1. Flavonoids as Natural Product of Anti-Virulence Agents

Flavonoids represent a viable natural product solution to the treatment of bacterial diseases by disarming potent virulence factors produced by the associated pathogens. These highly-conjugated compounds function in plants in many ways, including UV filtration and protection, nitrogen fixation (symbiosis), and floral pigmentation [79]. Many also serve to provide natural protection to the producing plant against microbes [79]. Quercetin is a well-known flavonoid found in numerous plant products, such as red onion, kale, grains, and fruits and vegetables. It has been shown to possess potent antibacterial activity against food-borne pathogens, such as *Escherichia coli*, *Staphylococcus aureus*, and *Pseudomonas aeruginosa* [96]. The eight flavonoid compounds that showed strong inhibitor activity against Plx2A (Table 1) are now prime candidates for further development, using the anti-virulence development pipeline shown in Figure 2. Previously, two compounds in this inhibitor series along with the synthetic M3 inhibitor were shown to possess good efficacy in model insect cell cultures but failed to protect honey bee larva from *P. larvae* infection [9]. The remaining flavonoids will be flowed through the development pipeline, including testing in the hive environment. Simultaneously, work will focus on improving the compounds’ potency through SARs/QSARs methods, which require high-resolution crystal structures with Plx2A and other C3-like toxins. This structural biology work is currently being pursued. Intriguingly, bio-active compounds such as Quercetin have the potential to act as double-edged swords against bacterial pathogens i.e., both as antibiotics and as anti-virulence compounds, to either kill pathogens or neutralize their weaponry, or a combination of both strategies. This attribute of antibiotic/anti-virulence flavonoids has appeal in the treatment of honeybee diseases such as American Foulbrood caused by the Gram-positive, soil-dwelling pathogen, *P. larvae*.

### 4.2. Inhibition of C3-like mART Toxins

We previously showed that the M3 synthetic inhibitor of C3larvin GH activity showed a different mechanism than has been observed for known mART inhibitors [60]. M3 is also a potent inhibitor of Plx2A GH activity [9]. An initial screen for inhibitors against C3larvin using our in-house libraries of mART inhibitors did not produce any hits, indicating that C3larvin and Plx2A (and the C3-like subgroup) possess a unique architecture associated with the ADP-ribosyltransferase (ADPRT) fold that requires a new, unique lead parent compound. Synthetic inhibitors, M2 and M3, satisfied those criteria. In the present study, we found that certain flavonoids, such as Quercetin, also inhibit Plx2A GH activity, and surprisingly, do not show competitive inhibition against the NAD^+^ substrate of Plx2A (Figure 5A), but rather exhibit a non-competitive mechanism often observed for allosteric modulators of regulatory enzymes. These results imply that flavonoids bind outside the NAD^+^-binding pocket of these C3-like toxins. Unfortunately, the specific binding site has not yet been identified since neither C3larvin nor Plx2A have formed diffraction-quality crystals in the presence of any of these flavonoid inhibitors. Remarkably, a survey of the binding surfaces of both C3larvin (PDB:4TR5; 5DZQ) and Plx2A (PDB:5URP) structures has failed to reveal a putative binding pocket for flavonoids and related inhibitor compounds, and it may be a case of an “induced” fit mechanism.

### 4.3. Anti-Virulence Strategy against American Foulbrood

Recently, it was shown that M3, Acacetin, and Baicalein compounds were strong inhibitors against Plx2A GH activity and serve as prospective compounds for an anti-virulence strategy to treat American Foulbrood in honey bees [9]. These three inhibitors were dosed into a model insect cell culture, where it was found that both M3 and Acacetin, but not Baicalein, protected the cultured cells from Plx2A intoxication [9]. M3 prevented the formation of bi-nucleated cells against Plx2A cytotoxicity at 300 µmol/L, whereas Acacetin protected the cell culture at 30 µmol/L. Unfortunately, when the anti-virulence compounds were administered to *P. larvae* ERIC I-infected honey bee larva, none of the Plx2A inhibitors reduced larval mortality and hence failed to provide protection against American Foulbrood disease. Several factors may be responsible for this lack of protection and further experiments are required to reveal the cellular mechanistic forces at work. One consideration for the failure of the inhibitors to protect larva is that Plx2A is not the only virulence factor produced by *P. larvae*, but is one of several that are responsible for the disease progression. Another possible explanation for the lack of an inhibitor effect in protecting larva may lie in the conditions found in the larval gut, which are quite complex. Perhaps the inhibitor compounds were unstable in the pH of the gut or digestive enzymes may have damaged the compounds, rendering them inactive. Further study is needed to reveal the complex factors that likely interplay in *P. larvae-*infected larva in the hive environment. Clearly these results suggest that treatment strategies involving host-pathogen interactions are complex and that a simple anti-virulence approach involving a single virulence factor such as Plx2A may prove insufficient to control the pathogen and its associated disease. Future work in this area will necessitate multi-level experiments that may involve a cocktail of anti-virulence agents to block the suite of virulence factors known to be active upon *P. larvae* infection of honey bee larva.

## 5. Conclusions

The anti-virulence or anti-infective approach shows great promise as an alternative to antibiotic therapy and may help alleviate the antibiotic resistance dilemma by targeting non-essential components of the bacterial pathogen, reducing the selective pressure on the organism to mutate or evolve. Natural products such as flavonoids have a bright future as potential agents for anti-virulence strategies for treating microbial pathogens. Remarkably, many of these plant-based compounds also show antibacterial activity in addition to their ability to block or curtail virulence factor function. Regardless, the anti-virulence approach is multi-faceted and requires a large funding base to execute the necessary steps to develop an anti-virulence drug. Academic research laboratories and institutions are not endowed with sufficient funds to meet this obligation. However, such organizations have shown the ability to produce good lead compounds for further development. The second level of anti-virulence agent development requires the intervention of industry, such as the pharmaceutical or agricultural sectors. Importantly, government involvement is further needed to help pave the road to success in this arena. Clearly, antibiotic development does not form the basis for a sound business model because of the resistance mechanisms intrinsic to the bacterial pathogens, and a paradigm shift is paramount. Notably, it is envisioned that anti-virulence compounds will not suffer this same fate, at least not to the same extent. However, this field is still embryonic and requires further research investment and execution to determine whether anti-virulence therapy becomes an important pillar to bacterial disease treatment.

## Figures and Tables

**Table 1:** Chemical Inhibitors of Plx2A.

Inhibitor	Chemical Name	Structure	^a^ K_D_ (μmol/L)	^b^ IC_50_ (μmol/L)
Acacetin	5,7-dihydroxy-2-(4-methoxy phenyl)-4H-chromen-4-one	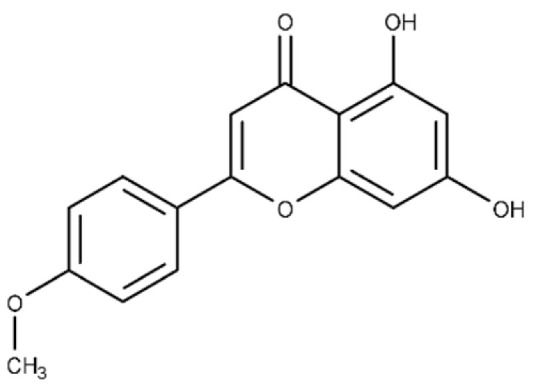	^c^ ND	28.0 ± 2.3
Baicalein	5,6,7-trihydroxy-2-phenyl-4H-chromen-4-one	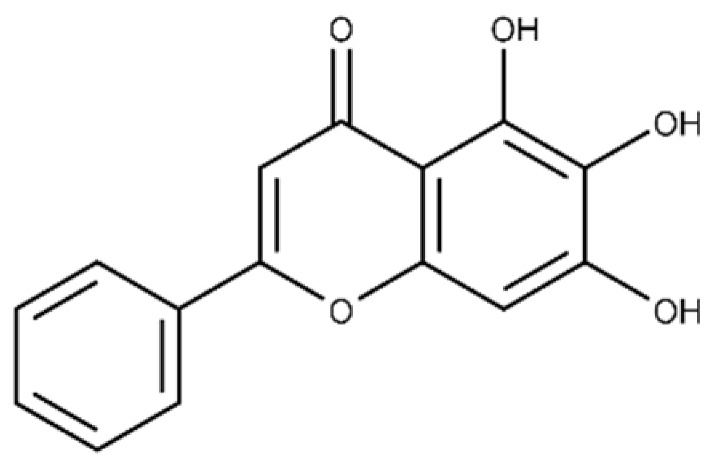	13.6 ± 1.5	10.7 ± 0.7
Chrysin	5,7-dihydroxy-2-phenyl-4H-chromen-4-one	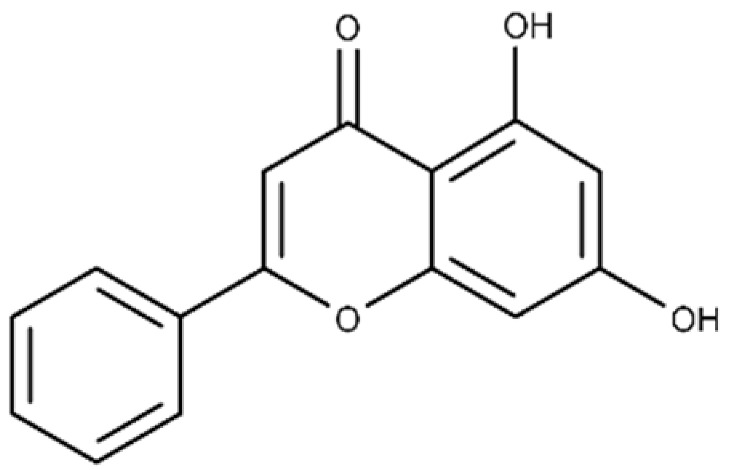	40.6 ± 4.7	49.2 ± 2.5
Jaceosidin	5,7-dihydroxy-2-(4-hydroxy-3-methoxyphenyl)-6-methoxy-4H-chromen-4-one	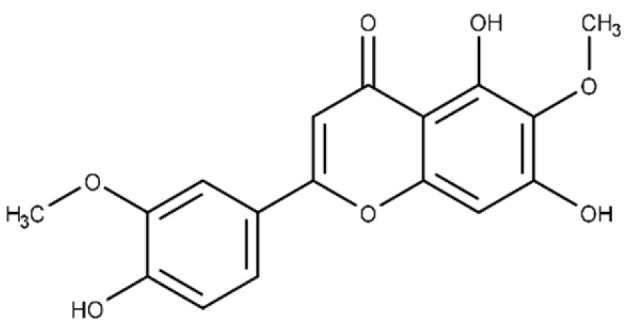	24.1 ± 2.9	73.9 ± 4.7
Kaempferol	3,5,7-trihydroxy-2-(4-hydroxyphenyl)-4H-chromen-4-one	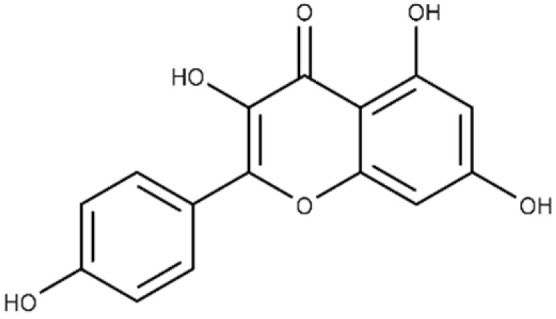	37.3 ± 3.6	79.9 ± 6.0
Luteolin	2-(3,4-dihydroxyphenyl)-5,7-dihydroxy-4H-chromen-4-one	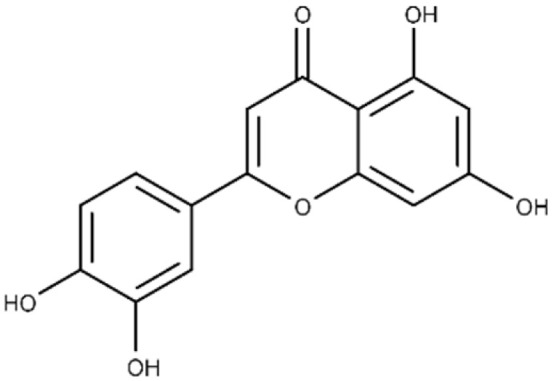	24.0 ± 1.5	68.1 ± 1.8
Morin	2-(2,4-dihydroxyphenyl)-3,5,7-trihydroxy-4H-chromen-4-one	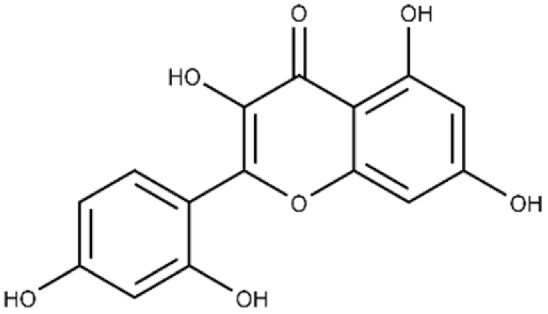	32.8 ± 11.7	109 ± 4.8
Quercetin	2-(3,4-dihydroxyphenyl)-3,5,7-trihydroxy-4H-chromen-4-one	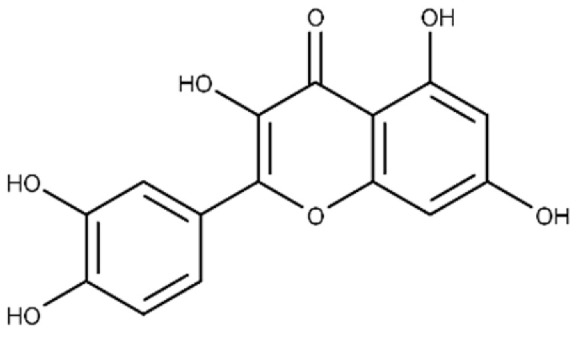	19.9 ± 0.4	24.3 ± 0.8
M3	N-{[(3R)-1-{1H-pyrazolo[3,4-d]pyrimidin-4- yl}piperidin-3-yl]methyl}methanesulfon amide	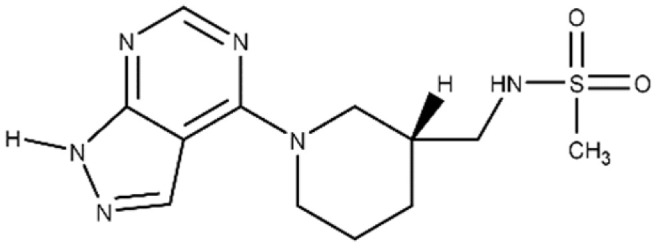	^c^ ND	216.3 ± 22.7

^a^ The binding affinity of inhibitors to Plx2A (*K*_D_) were calculated from the binding isotherms generated from the quenching of intrinsic Trp fluorescence of the protein upon titration with the inhibitor (ligand). The values are the mean ± S.D. from three separate experiments. ^b^ The IC_50_ values were calculated from the fit to a dose–response curve of Plx2A GH activity against the concentration of inhibitor/flavonoid compounds to produce a directed library of 20 compounds for experimental testing against Plx2A activity [9]. ^c^ ND, not determined.

## Data Availability

Data are available upon request, please contact the contributing authors.

## Abbreviations

ED_50_: effective concentration of inhibitor that reduces the toxic effect to 50%; ε-NAD^+^, etheno-NAD^+^; GH, glycohydrolase; IC_50_, inhibitor concentration that reduces the enzyme activity to 50%; mART, mono-ADP-ribosyltransferase; NATA, N-acetyltryptophanamide; SARs, structure–activity relationships; QSARs, quantitative structure–activity relationships; TD_50_, dose of the inhibitor/drug that causes toxic effects in 50% of the cells.
